# Detection of SARS-CoV-2 on the environmental surfaces and its implications for pandemic preparedness

**DOI:** 10.3389/fpubh.2024.1396334

**Published:** 2024-09-10

**Authors:** Kazi Jamil, Nasreem Abdulrazack, Saja Fakhraldeen, Heba Kamal, Anwar Al-Mutairi, Batool Al-Feili, Imtiaz Ahmed, Vinod Kumar

**Affiliations:** Environment and Life Sciences Research Center, Kuwait Institute for Scientific Research, Safat, Kuwait

**Keywords:** SARS-CoV-2, coronavirus, pandemic preparedness, environmental monitoring, surface sampling, RT-qPCR

## Abstract

Even though death due to COVID-19 is no longer a public health emergency, less virulent but highly transmissible forms of SARS-CoV-2 continue to spread in many countries leading to outbreaks and rise in hospitalizations in the affected regions. Lessons learned during the pandemic must be put into action to protect the world's population from another catastrophe like COVID-19. Novel approaches that were developed for tracking the spread of SARS-CoV-2 included analysis of wastewater, air samples, and various environmental surfaces. We conducted a study in Kuwait during the peak of COVID-19 pandemic to examine if SARS-CoV-2 could be detected in swabs taken from frequently touched environmental surfaces. We selected 12 Cooperative Society Stores—two from each governorate of Kuwait—for collection of surface samples. The Cooperative Society Stores are widely distributed across the whole country and cater to daily household needs including groceries and other essential items. These stores operated even during the “lockdown” imposed at the height of the pandemic. We collected swabs from high-touch surfaces including the handles of the shopping carts and freezers, the elevators, the keypads of the point-of-service terminals of cash counters, and the automated teller machines. All the surfaces tested showed a variable presence of SARS-CoV-2 by reverse transcriptase quantitative PCR, showing the validity of the proof-of-concept study. Monitoring of the presence of SARS-CoV-2 by surface sampling thus offers a cheap but effective means of environmental surveillance for coronaviruses. We therefore strongly recommend the addition of surface environmental sampling as a strategy for pandemic preparedness everywhere.

## Introduction

More than seven million lives were lost to COVID-19 since SARS-CoV-2 was first detected in December 2019 (1). Despite the availability of highly advanced technologies for detection of pathogens of pandemic potentials, the most developed countries of the world could not protect their population from the devastating consequences of the pandemic. While the virus continues to spread in a less virulent form, the governments face the challenge of producing the best possible strategy to prevent the next outbreak of the so-called “Disease X” (2). One Health approach has been proposed to integrate many diverse levels of infectious disease surveillance for early detection and efficient management of threats posed by the emerging and re-emerging pathogens of pandemic potential (3–6). The role of an Intergovernmental Panel for One Health for strengthening pandemic preparedness has also been critically evaluated by health policy experts (7). This paper presents the findings of a proof-of-concept study carried out in Kuwait during the COVID-19 pandemic for detection of SARS-CoV-2 on the environmental surfaces and discusses the implications of this approach for surveillance of emerging and re-emerging viral outbreaks.

Coronaviruses are important human and animal pathogens that may have originated in bats (8, 9). The major symptoms of COVID-19 were dry cough, sore-throat, fever, and various degrees of respiratory difficulties (10). The alarming rate of the spread of COVID-19 across the globe prompted many governments to take stringent isolation strategies including complete lock down of affected regions with closure of schools and offices. Even curfews have been implemented in many countries including Kuwait to restrict the mobility of the people and limit person-to-person spread. Human-to-human transmission of SARS-CoV-2 has already been confirmed (11). The major route of transmission of the virus is thought to be via respiratory droplets of infected humans, while transmission from contaminated inanimate surfaces to humans has also been documented (12–15). The virus has been detected in throat swab, blood, urine, and stool of infected patients by reverse-transcriptase quantitative PCR (RT-qPCR), and the viral RNA was detectable up to 25 days after the onset of symptom (12). The possibility of asymptomatic healthy people carrying the virus raises concerns about transmission in the community although the significance of this mode of transmission is unknown. WHO recommends frequent hand washing with soap and water and the use of face mask and personal protective equipment (PPE) to protect individuals who have the risk of exposure.

Previous studies on the persistence of human and veterinary coronaviruses on inanimate surfaces revealed that human coronaviruses such as SARS coronavirus, MERS coronavirus or endemic human coronaviruses can persist on inanimate surfaces like metal, glass, or plastic for up to 9 days (14). A controlled experimental study showed that SARS-CoV-2 may survive in the air for < 4 h while it may remain viable for 2 days on plastic and 3 days on metallic surface (15). It is unknown how long the virus remains viable in the different environments that exist in different countries with diverse climatic conditions where the virus continues to spread. The present study was conducted to detect the virus in the environment of Kuwait when the disease activity was already established in the community.

## Materials and methods

### Ethics statement

All the samples collected and analyzed in this study were obtained from environmental surfaces only. Although the viral RNA that were isolated from the surface samples most likely originated from humans, we did not collect any samples directly from any identifiable human subject. The study was, therefore, exempted from obtaining ethical approval or informed consent. However, the investigators considered the significant health risks posed to the research team while conducting this study, and obtained approval from the Ministry of Health of Kuwait to conduct the study although it was not a human subject research that required ethical approval and informed consent.

### Sample collection

**Figure 1:**
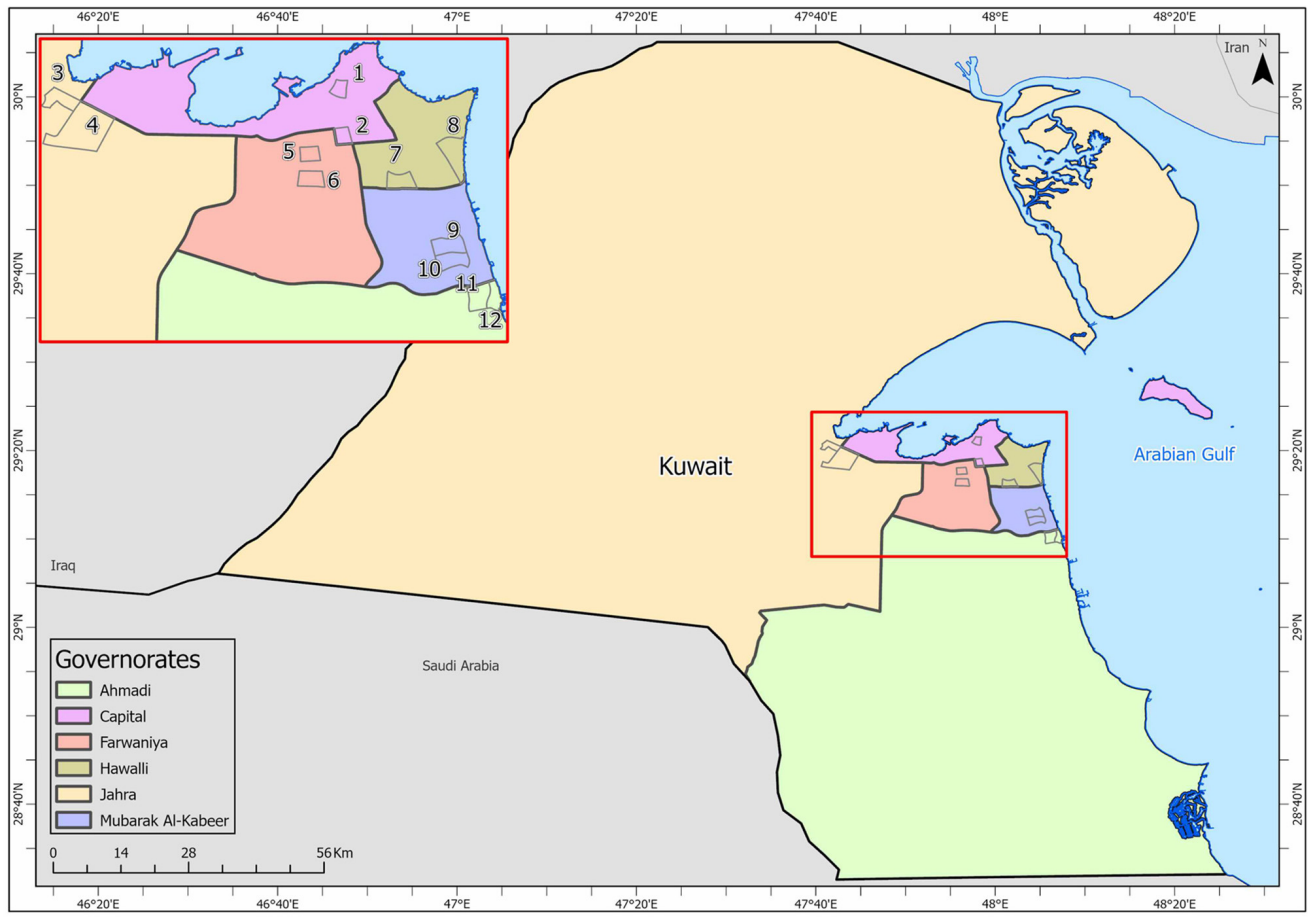
Kuwait map showing the sampling sites (Nos. 1–12) in the six governorates: Shamiya (1) & Yarmouk (2) in Capital Governorate; Qasr (3) & Saad Al-Abdullah (4) in Jahra Governorate; Rabiyah (5) & Ishbilia (6) in Farwaniya Governorate; Shuhada (7) & Salwa (8) in Hawalli Governorate; Al Qusour (9) & Qurain (10) in Mubarak Al Kabeer Governorate; Egaila (11) & Fintas (12) in Al Ahmadi Governorate.

Samples were collected from high-touch surfaces from twelve main Cooperative Society Stores, also known as Co-ops—two from each governorate of Kuwait—between June 6 and 23 of 2021 (Figure 1). The types of surfaces included building entrance doors outside the store and shopping cart handles, freezer door handles, keypads of automatic teller machines (ATMs), keypads of the point-of-service (POS) terminals of cash counters and elevator buttons inside the store. Sterile cotton-tipped swabs saturated with 1X PBS were used for sampling. Samples were collected from 25 cm^2^ of the surface by gently swabbing the entire area horizontally and vertically with uniform movement and occasionally rotating the swab for uniform surface coverage (16, 17). The surfaces used for collection of samples from the cooperative stores included the handles of the shopping carts (*n* = 159) and fridges (*n* = 32), the buttons of the elevators (*n* = 12), the keypads of the POS terminals of cash counters (*n* = 44), and the ATM machines (*n* = 22) located inside or adjacent to the same store. A few stores had an outside door with handles that were frequently used by the clients—therefore swab samples were collected from these door handles (*n* = 3) as well. In total, 272 swab samples were collected from the selected cooperative stores. One sample was lost in transport and 271 were analyzed by reverse transcriptase-quantitative PCR (RT-qPCR) as described below. In order to maximize the chances of detection of the virus, most of the samples (95.6%) collected from similar types of surfaces were pooled by combining 2–4 samples while < 5% of the samples were analyzed without pooling as shown in Tables 2–7. The total number of DNA samples actually analyzed by RT-qPCR was 83, denoted by “n^p^,” whereas the actual number of samples collected and processed was 271, denoted by “n.”

### Extraction of nucleic acids and RT-qPCR analysis

The swabs were dipped in 1 ml of TRIzol LS solution (Invitrogen, USA), and the RNA was isolated using the standard procedure described in the instruction manual (18). The total RNA was eluted in 10 μl of RNAse free water. All the steps were performed in BSL2 cabinet (Telstar BioII Advance Class II cabinet, Spain). Pooled samples were prepared by combining 5 μl aliquots of eluted RNA from 4 independent replicates of the same type of solid surface. A previously validated SARS-CoV-2 RT-qPCR protocol following the CDC guidelines and primers was used (16, 17, 19). RNA samples (2 μl) were converted to complementary DNA (cDNA) using iScript Reverse Transcription Supermix (BioRad, USA). The reverse transcription was carried out using random primers for 20 min at 46°C after priming for 5 min at 20°C. The reverse transcriptase enzyme was inactivated by incubating the reaction mix at 95°C for 1 min. The qPCR assays were performed on RNA samples to detect and quantify the SARS-CoV-2. A total of 6 serial dilutions ranging from 10^1^ to 10^6^ copies was prepared with a non-infectious synthetic lyophilized cDNA target encoding N gene and ORF1ab gene (Viasure SARS-CoV-2 positive control, CerTest Biotec S.L., Spain). This standard curve was used to determine the abundance of viral particles in RNA samples isolated from the surface swabs. The data was analyzed using the QuantStudio 5 software (Applied Biosystems, USA) by interpolating the absolute quantity of target in the test samples using the standard curve.

The cDNA (2 μl) was added to the reaction mixture with the forward primer 5′-GGGGAACTTCTCCTGCTAGAAT-3′ and reverse primer 5′-CAGACATTTTGCTCTCAAGCTG-3′ to amplify the N gene target of SARS-CoV-2 virus (15, 16). The total reaction volume was made up to 18μl with iTaq Universal SYBR Green Supermix, Forward and reverse primers, nuclease-free water, and 2 μl of sample cDNA was added. The PCR 20 μl reaction mix was loaded on the QuantStudio 5 Real-Time PCR System (Applied Biosystems, Singapore). Thermal cycling was performed at 95°C for 2 min and 40 cycles of 95°C for 5 s, 59.9°C for 1 min in QuantStudio^®^ 3 Applied Biosystems™ (Applied Biosystems, USA). The above-mentioned positive control from VIASURE SARS-CoV- Real-Time PCR Detection Kit-CE- IVD (Certest-Biotec, Spain) was used to validate the qPCR reactions. In addition, a known concentration of non-infectious synthetic construct of cDNA (2,000 copies/μl) comprising the N gene and ORF1ab genes of SARS-CoV- 2 were used as a positive control. The Ct values of the positive samples were quantified using the QuantStudio Design and Analysis software V1.5.2. (Applied Biosystems, USA).

## Results

**Figure 2:**
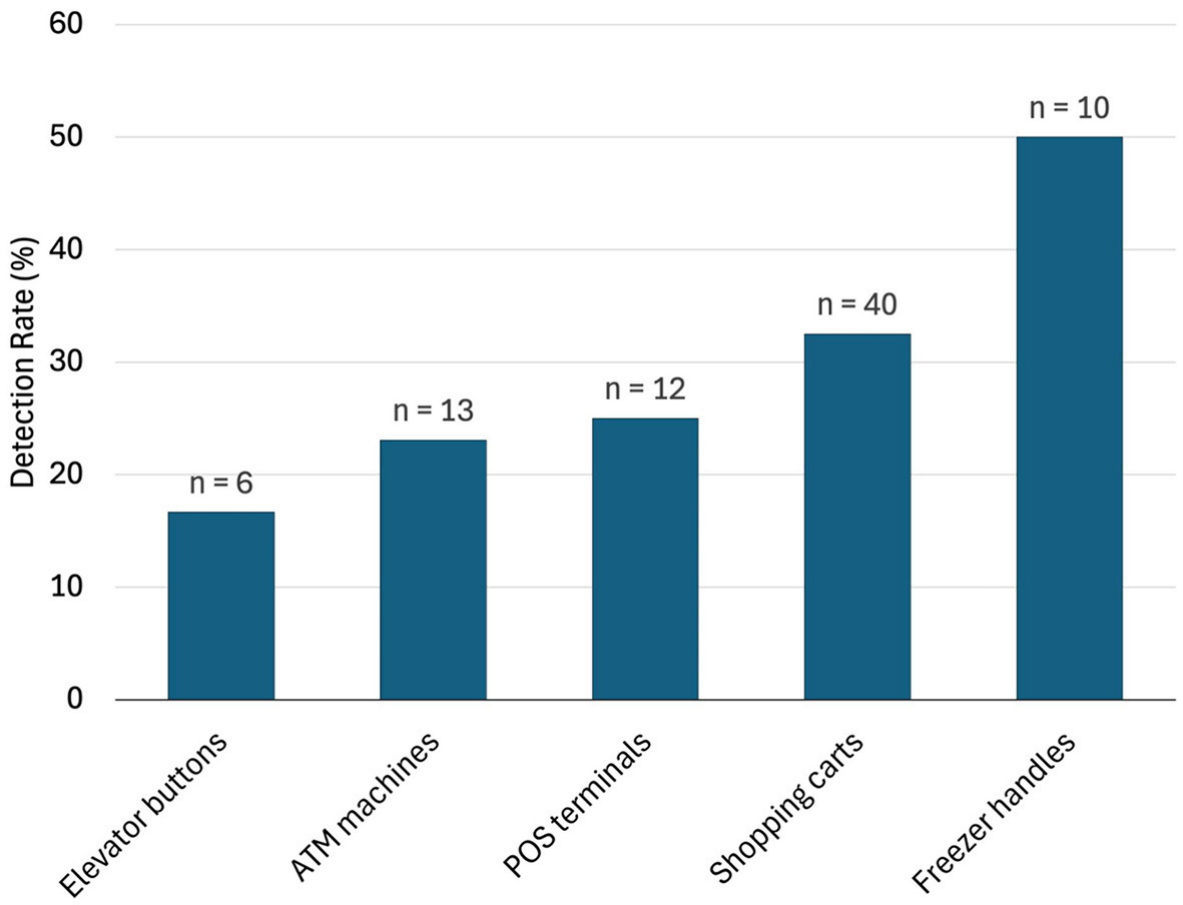
Rate of detection (percent) of SARS-Co-V-2 in pooled samples (n^P^ = 83) collected from the different types of surfaces (data from the outside door handles were all negative and not shown here).

In pooled samples analyzed by RT-qPCR (n^p^ = 83), the rate of positive test was highest with the freezer handles (50%) followed by the shopping carts (32.5%), the POS terminals (25%), ATMs (23%), and the elevators (16.66%) (Table 1, Figure 2). Tables 2–7 show the results of the RT-qPCR performed in the surface samples collected from all the cooperative societies. The samples were numbered according to the sequence of collection which was scheduled in coordination with the respective Cooperative Society Stores. Thus, the two stores of the same governorate were not always sampled in the same week as indicated by the differences in the sample numbers.

**Table 1:** Results of RT-qPCR performed on pooled samples (n^P^ = 83) collected from the surfaces of various objects at the study sites.

	**Objects tested by RT-qPCR**	**No. of pooled samples tested**	**No. of positive samples**	**No. of negative samples**	**Percentage of positive samples**	**Percentage of negative samples**
1	Shopping carts (handles)	40	13	27	32.5	67.5
2	ATM machines (keypad)	13	3	10	23.07	76.92
3	POS terminals at cash counter (keypad)	12	3	9	25	75
4	Elevator buttons	6	1	5	16.66	83.33
5	Freezer (handles)	10	5	5	50	50
6	Outside door handle	2	0	2	0	100

**Table 2:** Detection of SARS-CoV-2 in the surface samples collected from Al-Asimah governorate.

	**Sample nos**.	**Objects tested**	**PCR test result**	**CT value**	**Quantity**
Cooperative society store no. 1	1–4	Shopping carts (handle)	Positive	38.090	1.972E+01
	5–8	Shopping carts (handle)	Positive	36.656	5.394E+01
	9–12	Shopping carts (handle)	Positive	35.388	1.313E+02
	13–16	Shopping carts (handle)	Positive	31.740	1.695E+03
	17^*^	ATM machine (keypad)	NA	NA	NA
	18–20	ATM machines (keypad)	Positive	36.085	8.051E+01
	21–24	POS terminals at cash counter (keypad)	Negative	ND	ND
Cooperative society store no. 2	25–28	Shopping carts (handle)	Negative	ND	ND
	29–32	Shopping carts (handle)	Negative	ND	ND
	33–36	Shopping carts (handle)	Negative	ND	ND
	37–40	Shopping carts (handle)	Negative	ND	ND
	41–42	ATM machines (keypad)	Negative	ND	ND
	43–44	ATM machines (keypad)	Negative	ND	ND
	45–48	POS terminals at cash counter (keypad)	Negative	ND	ND

^*^Sample No. 17 was lost during processing and could not be analyzed. NA, not available; ND, not detected.

**Table 3:** Detection of SARS-CoV-2 in the surface samples collected from Jahra governorate.

	**Sample nos**.	**Objects tested**	**PCR test result**	**CT value**	**Quantity**
Cooperative society store no. 3	97–99	POS terminals at cash counter (keypad)	Positive	36.410	6.41E+01
	100–103	Shopping carts (handle)	Negative	ND	ND
	104–107	Shopping carts (handle)	Negative	ND	ND
	108–111	Shopping carts (handle)	Positive	35.237	1.46E+02
	112–116	Shopping carts (handle)	Positive	34.964	1.77E+02
	117–119	Freezer (handles)	Negative	ND	ND
	120	ATM machines (keypad)	Positive	32.734	8.44E+02
Cooperative society store no. 4	121–123	ATM machines (keypad)	Negative	ND	ND
	124–127	Shopping carts (handle)	Negative	ND	ND
	128–131	Shopping carts (handle)	Positive	37.125	3.88E+01
	132–135	Shopping carts (handle)	Negative	ND	ND
	136–139	POS terminals at cash counter (keypad)	Negative	ND	ND
	140–144	Freezer (handles)	Positive	37.744	2.52E+01

ND, Not detected.

**Table 4:** Detection of SARS-CoV-2 in the surface samples collected from Al-Farwaniya governorate.

	**Sample nos**.	**Objects tested**	**PCR test result**	**CT value**	**Quantity**
Cooperative society store no. 5	241–244	Shopping carts (handle)	Positive	34.047	1.93E+02
	245–248	Shopping carts (handle)	Negative	ND	ND
	249–250	POS terminals at cash counter (keypad)	Negative	ND	ND
	251–254	Freezer (handles)	Negative	ND	ND
	255	Elevator buttons	Negative	ND	ND
	256	ATM machines (keypad)	Negative	ND	ND
Cooperative society store no. 6	257–260	Shopping carts (handle)	Negative	ND	ND
	261–264	Shopping carts (handle)	Negative	ND	ND
	265–266	POS terminals at cash counter (keypad)	Negative	ND	ND
	267–269	Elevator buttons	Negative	ND	ND
	270–271	ATM machines (keypad)	Negative	ND	ND
	272	Freezer (handles)	Negative	ND	ND

ND, Not detected.

**Table 5:** Detection of SARS-CoV-2 in the surface samples collected from Hawally governorate.

	**Sample nos**.	**Objects tested**	**PCR test result**	**CT value**	**Quantity**
Cooperative society store no. 7	145–148	Shopping carts (handle)	Negative	ND	ND
	149–152	Shopping carts (handle)	Negative	ND	ND
	153–156	Shopping carts (handle)	Negative	ND	ND
	157–160	Shopping carts (handle)	Negative	ND	ND
	161–164	POS terminals at cash counter (keypad)	Negative	ND	ND
	165	ATM machines (keypad)	Negative	ND	ND
	166	Freezer (handles)	Positive	33.265	5.82E+02
	167–168	Outside door (handle)	Negative	ND	ND
Cooperative society store no. 8	169–172	Shopping carts (handle)	Negative	ND	ND
	173–176	Shopping carts (handle)	Negative	ND	ND
	177–180	Shopping carts (handle)	Positive	32.334	1.12E+03
	181–184	POS terminals at cash counter (keypad)	Positive	38.097	1.96E+01
	185	ATM machines (keypad)	Negative	ND	ND
	186–189	Freezer (handles)	Positive	36.827	4.78E+01
	190	ATM machines (keypad)	Negative	ND	ND
	191	Elevator buttons	Negative	ND	ND
	192	Outside door (handle)	Negative	ND	ND

ND, Not detected.

**Table 6:** Detection of SARS-CoV-2 in the surface samples collected from Mubarak Al-Kabeer governorate.

	**Sample nos**.	**Objects tested**	**PCR test result**	**CT value**	**Quantity**
Cooperative society store no. 9	49–52	Shopping carts (handle)	Negative	ND	ND
		53–56	Shopping carts (handle)	Positive	31.802	1.62E+03
		57–60	Shopping carts (handle)	Negative	ND	ND
		61–64	Shopping carts (handle)	Negative	ND	ND
		65	ATM machine (keypad)	Negative	ND	ND
		66	Elevator buttons	Positive	37.879	2.29E+01
		67–68	Fridge (handles)	Positive	36.802	4.87E+01
		69–72	POS terminals at cash counter (keypad)	Negative	ND	ND
Cooperative society store no. 10	73–77	Freezer (handles)	Negative	ND	ND
		78–82	POS terminals at cash counter (keypad)	Negative	ND	ND
		83–86	Shopping carts (handle)	Negative	ND	ND
		87–90	Shopping carts (handle)	Positive	39.864	5.69E+00
		91–94	Shopping carts (handle)	Positive	38.768	1.23E+01
		95–96	Shopping carts (handle)	Negative	ND	ND

ND, Not detected.

**Table 7:** Detection of SARS-CoV-2 in the surface samples collected from Al-Ahmadi governorate.

	**Sample nos**.	**Objects tested**	**PCR test result**	**CT value**	**Quantity**
Cooperative society store no. 11	193–196	Shopping carts (handle)	Negative	ND	ND
		197–200	Shopping carts (handle)	Negative	ND	ND
		201–204	Shopping carts (handle)	Positive	38.420	7.69E+00
		205–208	Elevator buttons	Negative	ND	ND
		209–212	POS terminals at cash counter (keypad)	Positive	37.191	1.90E+01
		213–214	ATM machines (keypad)	Positive	36.942	2.28E+01
		215–216	Freezer (handles)	Negative	ND	ND
Cooperative society store no. 12	217–220	Shopping carts (handle)	Negative	ND	ND
		221–224	Shopping carts (handle)	Negative	ND	ND
		225–228	Shopping carts (handle)	Negative	ND	ND
		229–232	POS terminals at cash counter (keypad)	Negative	ND	ND
		233–237	Freezer (handles)	Positive	37.226	1.85E+01
		238–239	Elevator buttons	Negative	ND	ND
		240	ATM machines (keypad)	Negative	ND	ND

ND, Not detected.

## Discussion

Kuwait has a population of 4.8 million people of which one-third are Kuwaiti citizens and the remaining are foreign nationals (20). According to the World Bank development indicators, 100 per cent of Kuwait's population have an urban life (21). Kuwait is divided into six governorates or provinces namely, Al-Asimah, Jahra, Hawalli, Farwaniya, Mubarak Al-Kabeer, and Al-Ahmadi. In Kuwait, there are around 60 Cooperative Society Stores, also known as Co-Ops, which are public entities distributed across the six governorates. The Co-ops are the grocery and convenience stores that account for most of the retail trade in Kuwait. In order to save lives, Kuwait Government took stringent measures including complete lockdown, curfews, and international travel bans during the COVID-19 pandemic. The Co-Ops, however, remained in service during the whole period of the pandemic but required online appointments for access. Since most of the institutions including offices, schools, and most shopping malls have been shut down during the pandemic, the Co-Ops were chosen for collection of swab samples from high-touch surfaces with all precautions recommended by the Ministry of Health of Kuwait.

Active surveillance of any infectious disease is traditionally conducted by collecting appropriate body fluids (blood, urine, stool, saliva, throat swab, etc.) from an adequate number of exposed people. Conducting active surveillance for SARS-CoV-2 in the community was extremely risky because of high virulence of the coronavirus. On the other hand, collection of swab samples from the high-touch surfaces in the Co-ops for detection of SARS-CoV-2 was a much safer approach as compared with collection of nasopharyngeal swabs from the exposed community dwellers. Although previous studies have confirmed the presence and viability of coronaviruses on some environmental surfaces in hospitals and elsewhere, the abundance and detectability of SARS-CoV-2 on environmental surfaces within the convenience stores were unknown. The unique distribution of the Co-ops across the whole country offered the study team the opportunity for collecting surface samples from these locations that were visited by many people daily even during the height of the pandemic.

The surface samples collected from the Co-Ops represented all the governorates of Kuwait. Almost every type of high touch surfaces tested in this study showed the presence of SARS-CoV-2 in variable proportions. While the handles of the shopping carts and the freezer handles showed high rate of positivity, the handles of the outside door were negative although they were used by many people visiting the same store. A possible explanation of this could be that the lower temperature inside the air-conditioned store had facilitated the prolonged viability of the virus on those surfaces whereas the outside high temperature reaching up to 125°F in June might have resulted in rapid degradation of the viral RNA on the entrance door handles. The longer contact time with the shopping carts as compared with the other objects tested could have accounted for the higher rate of viral detection in the former. The rate of detection of the virus on the keypads of the POS terminals was lower than that on the ATM machines probably because the former were frequently cleaned with disinfectants. Also, many clients made contactless payment using their cards without making any physical contact with the keypads with the keypads of the POS terminals. Thus, avoidance of physical contact with contaminated surfaces by the customers and frequent sanitization of the keypads could have resulted in lesser frequency of occurrence of virus particles on these surfaces.

Several research groups have examined the occurrence of SARS-CoV-2 on various types of touch surfaces during the COVID-19 outbreak (14–16, 22–27). A meta-analysis of 22 studies by Kampf et al. (14) revealed that human coronaviruses such as SARS coronavirus, MERS coronavirus, or endemic human coronaviruses can persist on inanimate surfaces like metal, glass, or plastic for up to 9 days, but can be efficiently inactivated by surface disinfection procedures. Also, a high frequency of occurrence of SARS-CoV-2 RNA was found on stainless steel and plastic surfaces (22). The results of the study we report here clearly show that high-touch surfaces are prone to accumulate viral particles that are detectable by RT-qPCR which has a 100% specificity for detection of the infection in humans. The application of environmental sampling in infectious disease epidemiology has a long history. Wastewater-based epidemiology (WBE) has been used by the WHO for poliovirus monitoring since 2003 (28). However, its application for tracking community-level infection trends gained widespread popularity during the COVID-19 pandemic. The worldwide interest in the use of WBE underscores the potential of environmental surveillance to complement traditional epidemiological methods (29). The detection of viral RNA in wastewater, regardless of viability of the virus, provided crucial information for health regulators to implement life-saving measures and mitigate the viral spread within the community. The surface sampling method employed in our study fundamentally shared the same objective as wastewater-based epidemiology (WBE)—to assess the presence and prevalence of SARS-CoV-2 in the environment. The relative ease of swab sample collection and testing offers a powerful tool for environmental surveillance of SARS-CoV-2. While the role of surface transmission remains uncertain, detecting the virus on surfaces can effectively monitor human cases and carriers within a community, allowing governments to optimize resource allocation and manage the situation effectively. Moreover, environmental surface testing provides a unique opportunity to detect potential SARS-CoV-2 resurgences, enhancing preparedness for future outbreaks or pandemics. We strongly advocate incorporating this approach into the arsenal of preventative measures against a future pandemic.

## Data Availability

The raw data supporting the conclusions of this article will be made available by the authors, without undue reservation.
